# Formation of swift heavy ion tracks on a rutile TiO_2_ (001) surface^1^

**DOI:** 10.1107/S1600576716013704

**Published:** 2016-09-23

**Authors:** Marko Karlušić, Sigrid Bernstorff, Zdravko Siketić, Branko Šantić, Ivančica Bogdanović-Radović, Milko Jakšić, Marika Schleberger, Maja Buljan

**Affiliations:** a Ruđer Bošković Institute, Bijenička cesta 54, Zagreb, 10000, Croatia; b Elettra-Sincrotrone Trieste, SS 14 km 163.5, Basovizza, 34149, Italy; c Fakultät für Physik and CENIDE, Universität Duisburg-Essen, Lotharstrasse 1, Duisburg, 47048, Germany

**Keywords:** swift heavy ions, ion tracks, TiO_2_, rutile, grazing-incidence small-angle X-ray scattering, GISAXS, atomic force microscopy, elastic recoil detection analysis

## Abstract

Nanostructuring of surfaces and two-dimensional materials using swift heavy ions offers some unique possibilities owing to the deposition of a large amount of energy localized within a nanoscale volume surrounding the ion trajectory. To fully exploit this feature, the morphology of nanostructures formed after ion impact has to be known in detail. In the present work the response of a rutile TiO_2_ (001) surface to grazing-incidence swift heavy ion irradiation is investigated. Surface ion tracks with the well known intermittent inner structure were successfully produced using 23 MeV I ions. Samples irradiated with different ion fluences were investigated using atomic force microscopy and grazing-incidence small-angle X-ray scattering. With these two complementary approaches, a detailed description of the swift heavy ion impact sites, *i.e.* the ion tracks on the surface, can be obtained even for the case of multiple ion track overlap. In addition to the structural investigation of surface ion tracks, the change in stoichiometry of the rutile TiO_2_ (001) surface during swift heavy ion irradiation was monitored using *in situ* time-of-flight elastic recoil detection analysis, and a preferential loss of oxygen was found.

## Abstract 2

Formation of ion tracks on a rutile TiO_2_ (001) surface after exposure to swift heavy ions under grazing incidence is studied using atomic force microscopy, grazing-incidence small-angle X-ray scattering and *in situ* time-of-flight elastic recoil detection analysis.

## 1. Introduction   

Swift heavy ions (SHIs) have found widespread use in research and technology, for both materials analysis and modification. Having a kinetic energy in the MeV range and above, their usability now spans diverse fields such as hadron therapy, industrial production of track etched membranes and testing of electronic devices against single-event upsets (Toulemonde *et al.*, 2004 ▸). In all those cases, dense electronic excitation localized in the wake of the ion is an important property of the SHI–matter interaction. Intense heating of the material due to electron–phonon coupling can trigger melting in a nanoscale volume along the ion trajectory, which upon rapid resolidification yields permanent damage called an ion track. Usually, these ion tracks have distinct physical and chemical properties, different from the surrounding matrix, and hence they can be subjected to various post-irradiation treatments like etching and grafting. While the manipulation of ion tracks remains the basis for numerous applications like the production of track etched membranes or ion beam lithography, intact ion tracks offer the opportunity to study the basic mechanisms of SHI–matter interaction (Itoh *et al.*, 2009 ▸; Aumayr *et al.*, 2011 ▸; Toulemonde *et al.*, 2012 ▸).

There are two distinct approaches in ion track studies, namely direct and indirect ion track measurements. A typical example of a direct ion track measurement technique is transmission electron microscopy (TEM), while the most commonly used indirect technique is channeling Rutherford backscattering spectroscopy (RBS/c). In the latter, average track radii can be estimated by measuring the kinetics of damage accumulation as a function of applied SHI fluence (Toulemonde *et al.*, 2012 ▸). Studies of the surface response to the SHI impact are less diverse. Although different energy dissipation channels like emission of secondary electrons or sputtering can be used to monitor SHI interaction processes with surfaces, atomic force microscopy (AFM) constitutes the most widely used technique to investigate nanostructures at the surface after exposure to SHI beams (Aumayr *et al.*, 2011 ▸). This approach allows a detailed investigation of ion track morphology, but obtaining reliable statistical information remains a time-consuming process owing to the low data accumulation speed of scanning probe methods.

While the first small-angle X-ray scattering (SAXS) studies of ion tracks date back to the 1980s (Albrecht *et al.*, 1985 ▸; Semenyuk *et al.*, 1991 ▸), it was only recently that SAXS was established as a powerful technique for indirect measurement of ion tracks (Abu Saleh & Eyal, 2004 ▸, 2005*a* ▸; Eyal & Abu Saleh, 2007 ▸; Pépy *et al.*, 2007 ▸; Kluth *et al.*, 2008 ▸). The advantages of SAXS over other techniques are numerous: it does not require single-crystal samples like RBS/c, it can be used to study radiation-sensitive materials like LiF which are very difficult to analyse with TEM, and it can resolve very small differences in the density between ion tracks and the surrounding matrix, as the scattered intensity is proportional to the electron density difference between them. This last point makes SAXS a perfect tool for the investigation of ion tracks in amorphous materials. Hence, it was quickly adopted to study ion tracks in metallic glasses (Rodríguez *et al.*, 2012 ▸), amorphous germanium (Ridgway *et al.*, 2013 ▸) and amorphous silicon (Bierschenk *et al.*, 2013 ▸). Note that SAXS is also perfectly capable of analysing ion tracks in crystalline matrices, as shown by studies of ion tracks in LiF (Schwartz *et al.*, 1998 ▸; Trautmann *et al.*, 2000 ▸; Abu Saleh & Eyal, 2005*b* ▸, 2007 ▸) and in quartz (Afra *et al.*, 2013 ▸). Finally, the manipulation of ion tracks by etching (Pépy *et al.*, 2007 ▸; Cornelius *et al.*, 2010 ▸; Kuttich *et al.*, 2014 ▸) and annealing (Schauries *et al.*, 2013 ▸, 2016 ▸) can be studied and even monitored *in situ* (Afra *et al.*, 2014 ▸). Further information can be found in a recent review on advanced techniques for ion track studies (Zhang *et al.*, 2015 ▸).

Recently, SHI irradiation performed at grazing incidence sparked intense research after a chain-like morphology of ion tracks at the surface of SrTiO_3_ was discovered (Akcöltekin *et al.*, 2007 ▸, 2008 ▸, 2009 ▸; Karlušić *et al.*, 2010 ▸). This kind of ion track morphology was explained by an oscillating electron energy loss of the SHI when penetrating through crystal planes, thus periodically encountering regions with increased density of electrons. In the past few years, novel nanoscale features like grooves in SiC (Ochedowski *et al.*, 2014 ▸), nanoholes within ion tracks in GaN (Karlušić *et al.*, 2015 ▸), cratering in polymers (Papaléo *et al.*, 2008 ▸, 2015 ▸), conductive ion tracks on a CaF_2_ surface (Roll *et al.*, 2008 ▸) and a surprising susceptibility of graphene to grazing incidence SHI irradiation (Akcöltekin *et al.*, 2011 ▸; Ochedowski *et al.*, 2013 ▸, 2015 ▸) have demonstrated convincingly that grazing-incidence SHIs have a tremendous potential for nanostructuring of surfaces and two-dimensional materials.

We have used grazing-incidence small-angle X-ray scattering (GISAXS) extensively for studies of SHI-induced ordering of quantum dots (Buljan *et al.*, 2009 ▸, 2010 ▸, 2011 ▸; Bogdanović-Radović *et al.*, 2012 ▸; Buljan, Radić *et al.*, 2012 ▸) and we have also used it as a tool for ion track measurements (Buljan, Karlušić *et al.*, 2012 ▸). In our work on ion tracks in GaN (Karlušić *et al.*, 2015 ▸) we have introduced GISAXS as a tool to analyse ion tracks on the material surface (*i.e.* surface tracks) formed after grazing-incidence SHI irradiation. Of course, this phenomenon has to be investigated under grazing-incidence geometry since the surface tracks are formed at the material surface. To the best of our knowledge, GISAXS has been used to study SHI impact sites on a surface only once before (Schattat *et al.*, 2005 ▸), but in that case normal-incidence SHI irradiation geometry was used. In both studies (Schattat *et al.*, 2005 ▸; Karlušić *et al.*, 2015 ▸), the main advantage of GISAXS over AFM was demonstrated, namely the absence of AFM tip size effects that place significant constraints on the AFM results. Given the large amount of interest in surface modifications using SHI beams (Aumayr *et al.*, 2011 ▸) this presents a significant advance. Very recently, it was demonstrated that TEM can also be used to study nanoscale surface features at SHI impact sites (Ishikawa *et al.*, 2015 ▸), thus bypassing constraints imposed by the AFM tip size. Still, those two techniques should be viewed as complementary. While TEM can visualize individual SHI impact sites, GISAXS as an indirect ion track measurement technique offers the advantage of acquiring significant statistical information with just one measurement on a large ion track ensemble.

Rutile TiO_2_ is an important technological material which has been subjected to various studies using SHI beams so far. The most notable studies involved patterning of the rutile TiO_2_ surface using ion beam lithography (Nomura *et al.*, 2003 ▸; Awazu *et al.*, 2005 ▸; Sanz *et al.*, 2006 ▸, 2007 ▸, 2010 ▸; Jensen *et al.*, 2008 ▸). Ion track measurements in rutile TiO_2_ have been reported using TEM (Awazu *et al.*, 2006 ▸), RBS/c (Popok *et al.*, 2009 ▸; Rivera *et al.*, 2010 ▸) and AFM (Thevenard *et al.*, 2000 ▸; Canut *et al.*, 2004 ▸; Awazu *et al.*, 2006 ▸). All of these above-mentioned studies were performed under normal-incidence SHI irradiation, which is a standard procedure to obtain quantitative ion track data that can be used to advance theories describing ion track formation processes (Rivera *et al.*, 2010 ▸; Karlušić & Jakšić, 2012 ▸).

In the present study, we performed grazing-incidence SHI irradiation on flat rutile TiO_2_ (001) surfaces and investigated the resulting surface tracks by AFM and GISAXS. In this way we were able not only to investigate in detail ion tracks on the rutile surface but also to compare the capabilities of these two techniques. In addition, we used time-of-flight elastic recoil detection analysis (TOF-ERDA) to monitor possible stoichiometric changes of the rutile phase during the SHI irradiation.

## 2. Experimental   

Single crystals of rutile TiO_2_ (001) with epi-polished surfaces were purchased from Crystec GmbH (Germany). Before the experiment, some of the samples were checked with AFM and, typically, the r.m.s. roughness was found to be 0.2 nm, with the surface free of contaminants. SHI irradiation was performed using 23 MeV I^6+^ ions delivered by the 6 MV EN Tandem Van de Graaff accelerator located at the Ruđer Bošković Institute, Zagreb, Croatia. All samples were irradiated under the same grazing-incidence angle of 1.25 ± 0.25°. The applied SHI fluences yielded surface track densities in the range of 10^9^–10^11^ surface tracks per cm^2^. Irradiations were performed at room temperature, with the ion beam scanned in order to ensure homogenous irradiation of the samples.

Possible preferential element losses, *i.e.* stoichiometric changes of the sample surface, were monitored by a TOF-ERDA spectrometer (described in detail by Siketić *et al.*, 2008 ▸, 2015 ▸; Siketić, Bogdanović Radović & Jakšić, 2010 ▸; Siketić, Bogdanović Radović, Jakšić & Skukan, 2010 ▸). Measurements were performed using the same 23 MeV I^6+^ ions, at either 20 or 1° incidence angle toward the sample surface. All data were collected in the so-called ‘list mode’ (event-by-event detection), which enables an easy offline replay of the measurement. Offline analysis, with replay sections for the first 10 nm, was performed with the analysis software package *Potku* (Arstila *et al.*, 2014 ▸). Another simulation code, *SIMNRA*, was used for the calculation of the total number of ions hitting the sample, using the known solid angle of the TOF-ERDA spectrometer (Mayer, 1997 ▸).

**Figure 1:**
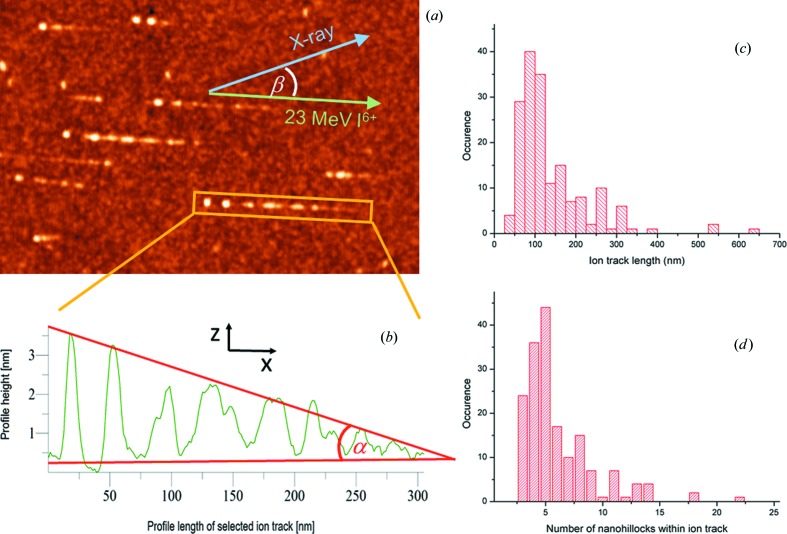
(*a*) AFM image of surface tracks on a rutile TiO_2_ (001) surface after exposure to 23 MeV I^6+^. The surface is irradiated with SHIs under grazing incidence (green arrow), giving rise to surface tracks aligned with the ion beam direction. The angle between the X-ray beam (blue arrow) for the GISAXS analysis and the ion beam is β. (*b*) Profile of a selected surface track with α denoting the tilt of the surface track. (*c*) Surface track length distribution for a SHI grazing-incidence angle of 1.25 ± 0.25°, determined from the analysis of 173 individual surface tracks. (*d*) Histogram showing the distribution of the number of nanohillocks within those surface tracks.

To investigate the topography of the surface after irradiation, *ex situ* tapping mode AFM was carried out using a Dimension 3100 instrument (Veeco Metrology, USA) and NCHR cantilevers (Nanosensors, Switzerland) with cantilever resonance frequencies of around 300 kHz. Images were reproduced by the *WSxM* code (Horcas *et al.*, 2007 ▸). The topography of the surface was also investigated using GISAXS, which was performed at Elettra-Sincrotrone Trieste, Italy, on the SAXS beamline (Amenitsch *et al.*, 1995 ▸). Samples were placed on a rotational stage that allows precise sample positioning, in order to measure sets of GISAXS spectra for different azimuthal angles β from each sample (see Fig. 1 ▸ *a*). During the exposure, a highly collimated beam of X-rays illuminated the sample surface at grazing-incidence angle. Each sample was measured for three grazing-incidence angles (critical angle of total reflection and two angles slightly above and below the critical angle). A two-dimensional Dectris detector was used to record the GISAXS intensity maps obtained from scattering of 8 keV photons (λ = 0.154 nm).

## 3. Results and discussion   

After exposure of the rutile TiO_2_ (001) surface to the grazing SHI irradiation, long chains of nanohillocks could be observed using AFM. Like in the case of SrTiO_3_ (Akcöltekin *et al.*, 2007 ▸, 2008 ▸; Karlušić *et al.*, 2010 ▸), several equally spaced nanohillocks can be found within each surface track, as shown in Fig. 1 ▸(*b*). These examples of surface track profiles reveal a striking periodicity within an ion track, and their potential for nanopatterning was recognized early on (Akcöltekin *et al.*, 2007 ▸). Surface tracks show a great diversity in different materials but they all have in common the feature that their properties can be controlled to some degree by the SHI irradiation parameters. While the heights of the nanohillocks show similar values, the average length of the ion track and the average distances between nanohillocks within the ion track can be tuned by the incident angle of the SHI beam (Akcöltekin *et al.*, 2008 ▸, 2009 ▸). The energy of the SHI beam also influences the length of the surface track (Karlušić *et al.*, 2010 ▸) and sometimes even the surface track morphology (Karlušić *et al.*, 2015 ▸). By fixing the SHI beam irradiation parameters, the surface track details can be inspected more closely. We define the length of the surface track as the distance between the top (determined from the maximum in a linescan across the hillock) of the first and the top of the last nanohillock within the surface track. The distributions of the measured surface track lengths and the number of nanohillocks within each surface track are shown in Figs. 1 ▸(*c*) and 1 ▸(*d*). The similarity of these two distributions provides evidence for a rather uniform distance between neighbouring nanohillocks within the ion track, which we estimate to be 26 ± 6 nm. The height of the nanohillocks ranges between 1 and 2 nm, but sometimes we observe the average height of the leading nanohillocks to exceed 3 nm, as shown in Fig. 1 ▸(*b*). Nanohillocks with a height below 0.5 nm were difficult to identify because the r.m.s. roughness of the unirradiated TiO_2_ surface is 0.2 nm, and were thus not considered for the analysis.

**Figure 2:**
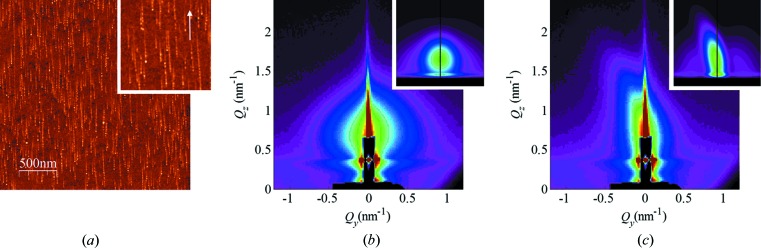
(*a*) AFM image of non-overlapping ion tracks on a rutile TiO_2_ (001) surface (50 ion tracks per µm^2^, image height scale 4 nm, inset ×2 magnification). GISAXS maps of the irradiated surface acquired at (*b*) β = 0° and (*c*) β = 5°. The corresponding simulations of the GISAXS maps are shown as insets in (*b*) and (*c*). The simulations are generated using the parameters of the fit.

**Figure 3:**
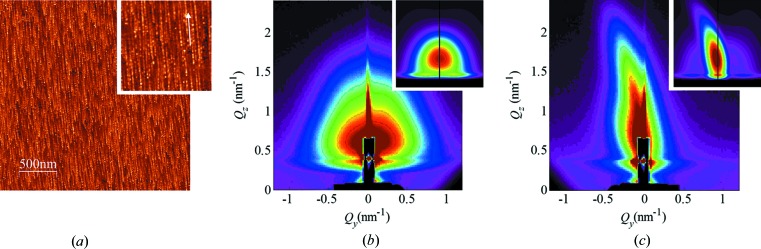
(*a*) AFM image of overlapping ion tracks on a rutile TiO_2_ (001) surface (250 ion tracks per µm^2^, image height scale 4 nm, inset ×2 magnification). GISAXS maps of the irradiated surface acquired at (*b*) β = 0° and (*c*) β = 5°. The corresponding simulations of the GISAXS maps are shown as insets in (*b*) and (*c*). The simulations are generated using the parameters of the fit.

**Figure 4:**
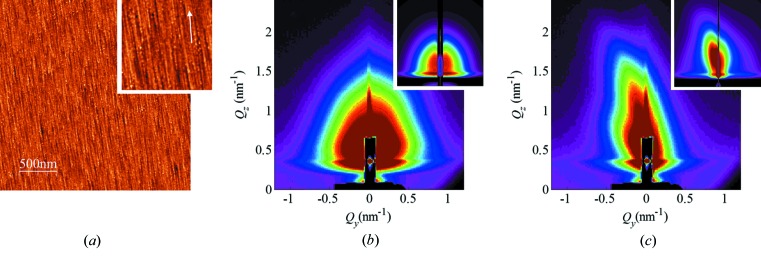
(*a*) AFM image of multiple overlapping ion tracks on a rutile TiO_2_ (001) surface (900 ion tracks per µm^2^, image height scale 4 nm, inset ×2 magnification). GISAXS maps of the irradiated surface acquired at (*b*) β = 0° and (*c*) β = 5°. The corresponding simulations of the GISAXS maps are shown as insets in (*b*) and (*c*). The simulations are generated using the parameters of the fit.

In order to achieve further progress in the analysis of surface tracks, here we employ the GISAXS technique. GISAXS offers an advantage in such a study because billions of surface tracks can be analysed simultaneously in a single sample; thus experimental data with excellent statistics can be acquired quickly. Another advantage of GISAXS over AFM is the absence of artefacts that are present in the AFM images, due to the finite size of the AFM tip. These artefacts limit the usability of AFM to the accurate measurement of the surface track length and height, the number of nanohillocks, and the distance between individual nanohillocks. Finally, GISAXS provides a reliable tool of analysis for surfaces irradiated with high SHI fluences, a feat that is notoriously difficult to achieve by means of AFM. Here we demonstrate (Figs. 2 ▸–4 ▸ ▸) the GISAXS capabilities by analysing a set of samples irradiated with different SHI fluences, but otherwise irradiated under identical conditions like angle of incidence and SHI energy. In these figures, as shown by the accompanying AFM images, the applied SHI fluence ranged from well separated, *i.e.* non-overlapping surface tracks (Fig. 2 ▸), to a surface completely covered by surface tracks (Fig. 3 ▸), and finally to multiple surface track overlap (Fig. 4 ▸).

**Figure 5:**
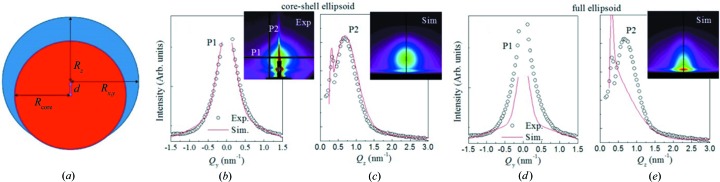
(*a*) Schematic of the model used for the description of nanoparticle shape. (*b*), (*c*) Intensity profiles of the GISAXS map shown in the inset of panel (*b*) taken along the lines indicated by P1 and P2, together with the intensity profiles of the simulated map obtained by a fit using the core/shell structure of nanohillocks shown in panel (*a*). (*d*), (*e*) Intensity profiles of the GISAXS map shown in the inset of panel (*b*) taken along the lines indicated by P1 and P2, together with the intensity profiles of the simulated map obtained by a fit using the full-ellipsoid structure of nanohillocks.

The GISAXS maps of the irradiated samples taken at the critical angle are shown in panels (*b*) and (*c*) of Figs. 2 ▸–4 ▸ ▸. Two characteristic patterns appear for all films, which depend on the orientation of the surface tracks with respect to the probing X-ray beam (angle β). When the beam is aligned parallel (β = 0°) to the surface tracks, a symmetric, nearly circular scattering pattern is visible [panels (*b*)]. The shapes of the patterns indicate that the formed nanohillocks are ellipsoids with core/shell internal structure. That follows from the strong intensity spots that are visible in the β = 0° maps. Such an intensity distribution, and especially form factor contribution, can be achieved only if some type of core/shell structure of the ellipsoid is assumed. More precisely, the shape of the nanohillock is assumed to be ellipsoidal (with radii *R*_*x*,*y*,*z*_) with a spherical core (having radius *R*_core_) that is shifted by the vector **d** from the ellipsoid origin. A schematic of a nanohillock cross section with such structure is shown in Fig. 5 ▸(*a*). Using this structure it is possible to get the fit similar to the measurement, as illustrated by the profiles shown in Figs. 5 ▸(*b*) and 5 ▸(*c*). Using a simple full ellipsoid cannot provide good agreement with the measurements (Figs. 5 ▸ *d* and 5 ▸ *e*). σ_R_ is the standard deviation of the size distribution. This roughly corresponds to the core covered by the surface layer of other material from the top. For details about core/shell structure see the article by Buljan *et al.* (2015 ▸), where the same shape model is used. The core/shell assumption may be supported by the fact that a significant amount of water is absorbed in the surface layer of the irradiated part of the surface (Popok *et al.*, 2009 ▸). It is also possible that two phases are formed, but a more detailed analysis of the internal structure of the nanohillocks will be the topic of our future work. When the probing X-ray beam is aligned with respect to the surface tracks with β = 5°, the typical scattering pattern has characteristic tails, as visible in panels (*c*) of Figs. 2 ▸–4 ▸ ▸. The intensity distribution of the tail is related to the length of the surface track and the position of the nanohillocks in it. We have performed a numerical analysis of the scattering patterns in the following way. We used a specially adopted paracrystal model for the analysis of the GISAXS intensity distributions (Buljan, Radić *et al.*, 2012 ▸; Buljan *et al.*, 2016 ▸). The nanohillocks are assumed to be arranged in chains characterized by basis vector **a**_1_ which is aligned along the formed tracks. We assume that the irradiation is performed in the *xz* plane (see Fig. 1 ▸ *b*). Thus, the nanohillocks have a separation |**a**_1_| within the track. The number of nanohillocks within the chain is *N*. The position of the *i*th nanohillock within the chain (**R** *_i_*) is given by **R** *_i_* = *i* **a**_1_ + **δ**_*x*_ + **δ** *_y_* + **δ** *_z_*. The vector components **δ**_*x*,*y*,*z*_ denote the deviation of the nanohillock from the ideal position. We assume a normal distribution of the deviation vectors **δ**_*x*,*y*,*z*_ from the ideal positions with the standard deviations σ_*x*,*y*,*z*_.

Additionally, for the high irradiation dose when ion tracks overlap (Fig. 4 ▸ *c*), we assume the existence of correlation in ion track separation. In addition to the parameters described for non-correlated tracks, this model assumes that all tracks are arranged in a short-range-ordered paracrystal lattice having separation |**a**_2_| and |**a**_3_| in the directions perpendicular and parallel to the ion beam directions, respectively. The deviations of the track positions are described by parameters 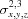 (for details see Buljan *et al.*, 2016 ▸). Details of the model are also given by Buljan *et al.* (2016 ▸). The simulations of the experimental data obtained by using the results of the numerical analysis are shown in the insets of the GISAXS maps in Figs. 2 ▸–4 ▸ ▸, while the results of the analysis are given in Table 1 ▸. As well as the values given there, the model describing overlapping tracks has additional parameters. They have the following values: |**a**_2_| = 22 ± 2 nm, |**a**_3_| = 170 ± 10 nm, 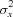 = 15 ± 2 nm, 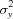 = 16 ± 2 nm, 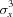 = 6 ± 1 nm, 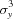 = 4 ± 1 nm, 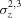 = 0.5 ± 0.1 nm. From the results it follows that the thickness of the shell layer is less than 6 Å, that is, only a few atomic layers. Such a thin surface layer could easily be the consequence of the interaction of the ion-treated surface with the surrounding atmosphere.

**Figure 6:**
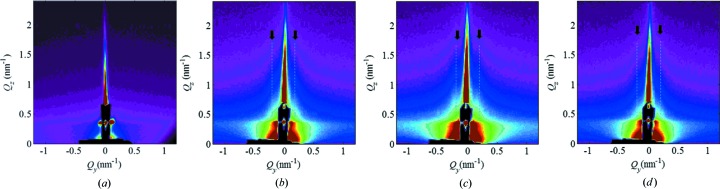
GISAXS maps of (*a*) the non-irradiated surface and surfaces irradiated with (*b*) 50, (*c*) 250 and (*d*) 900 ion tracks per µm^2^ obtained at β = 90°

Finally, the GISAXS measurements of the non-irradiated film and typical maps for the angle β = 90° are given in Fig. 6 ▸. As visible in Fig. 6 ▸, there is practically no scattered intensity for the non-irradiated film, except from the intrinsic surface roughness. The maps for the angle β = 90° shows two haracteristic vertical sheets at *Q_y_* = 0.21 nm^−1^ [indicated by dashed lines in Figs. 6 ▸(*b*)–6 ▸(*d*)]. Although these sheets are relatively weak, they are related to the correlation in the nanohillock positions within the tracks, and the characteristic separation |**a**_1_| is determined from their *Q_y_* positions. The fitting was not performed for these maps because of the very weak scattered signal. These sheets are most pronounced for the middle-fluence irradiated film. The high concentration of single-ion tracks is probably the reason for this. The track overlapping for the highest fluence is significantly larger, which destroys the regularity within the single-ion tracks.

GISAXS cannot resolve the contributions from the surface and from below the surface. Namely, the surface contribution is much stronger owing to the larger electron density contrast between air and the formed nanohillocks. Thus, the results of the structural analysis show the formation of nanohillocks that are well ordered within the surface ion tracks. The uniformity of the separations in the **x** direction (parallel to the tracks) is better for the lower-fluence films. The same is true for the quality of ordering in the **y** direction (perpendicular to the ion track). An increase of the fluence results in track overlapping, which induces disorder in the nanohillocks’ arrangement. Another effect of fluence increase and track overlapping is the growth of the nanohillocks’ radii in the direction parallel to the substrate (*R_xy_*). At the same time the vertical radius *R_z_* decreases (which is in accordance with the analysis of the AFM images) and the size distribution narrows.

**Figure 7:**
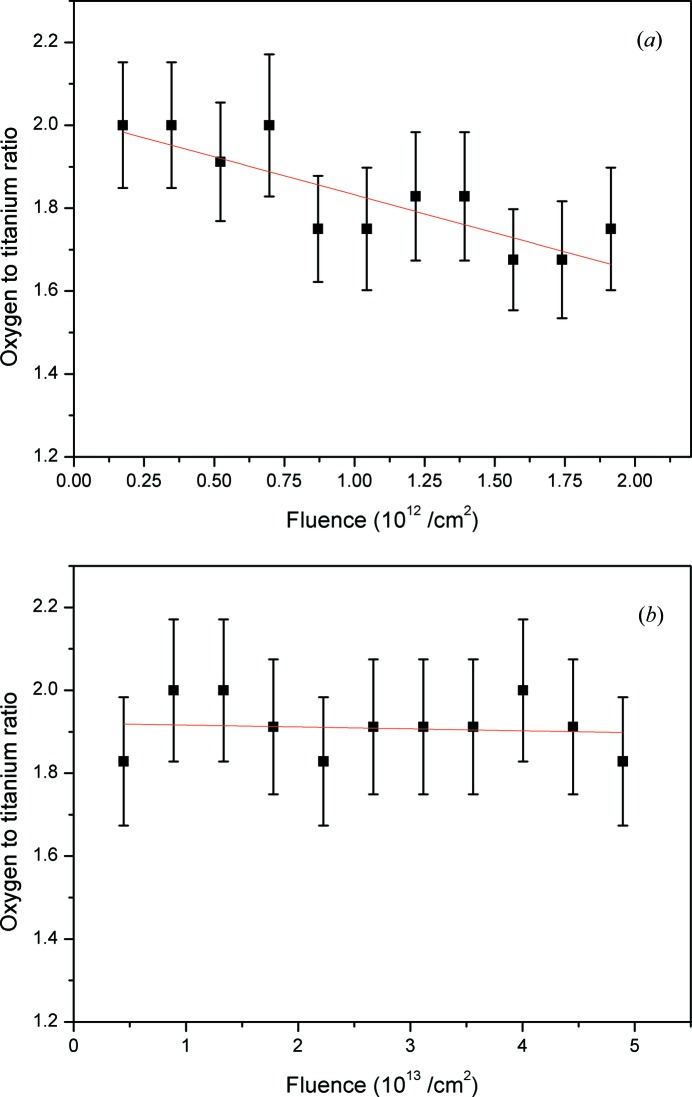
Preferential loss of oxygen (*a*) from the first 10 nm monitored by TOF-ERDA during grazing-incidence irradiation using 23 MeV I^6+^ ions. The stoichiometry of the surface remains the same (*b*) during TOF-ERDA measurement using the same ion beam but irradiated at 20° incidence angle. Red lines are linear fits to the data.

In addition to the structural analysis of the surface tracks, *in situ* TOF-ERDA was used to investigate possible stoichiometric changes during irradiation of rutile TiO_2_. The result is shown in Fig. 7 ▸(*a*) and indicates a pronounced loss of oxygen from the first 10 nm that accompanies the formation of surface tracks on rutile TiO_2_.

In our previous work on GaN (Karlušić *et al.*, 2015 ▸), we observed nanoholes within the surface tracks on the GaN surface which gave a clear indication of material removal during SHI irradiation. The appearance of these nanoholes coincides with a preferential loss of nitrogen also observed using *in situ* TOF-ERDA. To provide an explanation for the observation of both nanoholes on the GaN surface and preferential loss of nitrogen, the thermal decomposition of the GaN due to a thermal spike was invoked.

In the case of rutile TiO_2_, it is clear from the AFM images that the surface tracks consist of nanohillocks only. Even though it is not as pronounced as in the case of GaN, the preferential loss of oxygen from the first 10 nm as shown in Fig. 7 ▸(*a*) is unexpected. TOF-ERDA measurements using the same ion beam but performed under 20° incidence angle shown in Fig. 7 ▸(*b*) reveal that the oxygen to titanium ratio is stable within the first 20 nm. Since the preferential loss of oxygen occurs only under grazing-incidence SHI irradiation, similarly to the case of GaN, this suggests again that the process is driven by an oscillating electronic energy loss. In the case of grazing-incidence SHI irradiation, the peaks of the electronic energy loss can easily surpass its average value (which can be calculated by the *SRIM* code; Ziegler *et al.*, 2010 ▸) by a factor of two or more (Akcöltekin *et al.*, 2008 ▸), thus giving rise to an extremely localized melting and subsequent nanohillock formation. But melting alone is generally not considered sufficient for sputtering in the electronic energy loss regime and the vaporization criterion has to be met (Toulemonde *et al.*, 2002 ▸). An amorphization of the TiO_2_ surface due to multiple ion track overlap could promote an electronic sputtering process because it is known that amorphous materials are more sensitive to dense electronic excitations than their crystalline counterparts (Itoh *et al.*, 2009 ▸). But even under such conditions, previous results of ERDA on polycrystalline anatase TiO_2_ thin films using higher-energy 40 MeV I^9+^ ions did not yield any stoichiometric changes, although significant electronical sputtering seemed to take place (Jensen *et al.*, 2010 ▸).

Therefore, an explanation for the observed preferential loss of oxygen remains elusive at this point. Because of charge imbalance arising from a large number of secondary electrons ejected into the vacuum, perhaps Coulomb explosion could be opened up as another channel of SHI energy dissipation on the surface (Arnoldbik *et al.*, 2005 ▸; Karlušić & Jakšić, 2012 ▸) that could drive the observed sputtering. Whether this process is responsible for the oxygen depletion that accompanies surface track formation on the surface of rutile TiO_2_ (001) could be investigated by molecular dynamics simulations (Bringa & Johnson, 2002 ▸) but is beyond the scope of this work.

## 4. Conclusion   

In the present study, GISAXS and AFM were used to investigate surface tracks on a rutile TiO_2_ (001) surface formed after grazing-incidence SHI irradiation. We have shown that these two complementary techniques can be used successfully to extract detailed structural information about surface tracks in a wide range of irradiation fluences. It has been shown previously that the SHI’s energy and angle of incidence can be utilized to change the morphology of the surface track (Karlušić *et al.*, 2015 ▸, 2010 ▸; Akcöltekin *et al.*, 2008 ▸). Here we demonstrate how the applied SHI fluence can be used for nanoscale patterning of the surface. We have investigated three irradiation regimes, namely non-overlapping ion tracks, overlapping ion tracks and multiple overlapping ion tracks. The successful characterization of the surface in all three different irradiation regimes as presented here constitutes the first and necessary step for exploiting surface patterning by grazing-incidence SHI irradiation.

The preferential loss of oxygen from the rutile TiO_2_ (001) surface during grazing-incidence SHI irradiation, monitored by *in situ* TOF-ERDA, opens up again the question of the composition of surface tracks. This surprising result clearly warrants further studies.

## Figures and Tables

**Table 1:** GISAXS results for irradiated rutile TiO_2_ |**a**_1_| denotes the separation of the hillocks in the track, *N* is their mean number in the track, σ_*x*,*y*,*z*_ are the deviation parameters in the particular direction (*x*, *y*, *z*), *R*_*x*,*y*,*z*_ and *R*_core_ denote the total radii of the hillocks in the given directions and the core radius (the core radius is scaled to fit the ellipsoidal shape of the entire hillock), σ*_R_* is the standard deviation of the size distribution, and |**d** *|* is the absolute value of the shift of the core origin. Values in parentheses indicate the uncertainties on the least significant digit.

Track density (µm^−2^)	|**a**_1_| (nm)	*N* (nm)	σ*_x_* (nm)	σ_*y*_ (nm)	σ_*z*_ (nm)	*R*_*x*,*y*_ (nm)	*R_z_* (nm)	*R*_core_ (nm)	σ_*R*_ (nm)	|**d**| (nm)
50	29 (1)	6 (3)	10 (3)	2.2 (2)	0.26 (6)	1.9 (1)	1.9 (1)	1.6 (1)	1.1 (2)	0.39 (2)
250	29 (1)	5 (3)	10 (3)	1.4 (2)	0.5 (1)	2.2 (1)	1.8 (1)	1.5 (2)	1.0 (2)	0.41 (2)
900	29 (1)	6 (3)	10 (3)	1.1 (2)	0.5 (1)	2.3 (1)	1.7 (1)	1.5 (1)	1.0 (2)	0.55 (2)

## References

[bb51] Abu Saleh, S. & Eyal, Y. (2004). *Appl. Phys. Lett.* **85**, 2529–2531.

[bb52] Abu Saleh, S. & Eyal, Y. (2005*a*). *Nucl. Instrum. Methods Phys. Res. Sect. B*, **236**, 81–87.

[bb53] Abu Saleh, S. & Eyal, Y. (2005*b*). *Nucl. Instrum. Methods Phys. Res. Sect. B*, **230**, 246–250.

[bb1] Abu Saleh, S. & Eyal, Y. (2007). *J. Appl. Cryst.* **40**, s121–s125.

[bb2] Afra, B., Nordlund, K., Rodriguez, M. D., Bierschenk, T., Trautmann, C., Mudie, S. & Kluth, P. (2014). *Phys. Rev. B*, **90**, 224108.

[bb3] Afra, B., Rodriguez, M. D., Trautmann, C., Pakarinen, O. H., Djurabekova, F., Nordlund, K., Bierschenk, T., Giulian, R., Ridgway, M. C., Rizza, G., Kirby, N., Toulemonde, M. & Kluth, P. (2013). *J. Phys. Condens. Matter*, **25**, 045006. 10.1088/0953-8984/25/4/045006 23238277

[bb4] Akcöltekin, E., Akcöltekin, S., Osmani, O., Duvenbeck, A., Lebius, H. & Schleberger, M. (2008). *New J. Phys.* **10**, 053007.

[bb7] Akcöltekin, E., Peters, T., Meyer, R., Duvenbeck, A., Klusmann, M., Monnet, I., Lebius, H. & Schleberger, M. (2007). *Nat. Nanotechnol.* **2**, 290–294. 10.1038/nnano.2007.109 18654286

[bb5] Akcöltekin, S., Akcöltekin, E., Roll, T., Lebius, H. & Schleberger, M. (2009). *Nucl. Instrum. Methods Phys. Res. Sect. B*, **267**, 1386–1389.

[bb6] Akcöltekin, S., Bukowska, H., Peters, T., Osmani, O., Monnet, I., Alzaher, I., Ban d’Etat, B., Lebius, H. & Schleberger, M. (2011). *Appl. Phys. Lett.* **98**, 103103.

[bb8] Albrecht, D., Armbruster, P., Spohr, R., Roth, M., Schaupert, K. & Stuhrmann, K. (1985). *Appl. Phys. A*, **37**, 37–46.

[bb9] Amenitsch, H., Bernstorff, S. & Laggner, P. (1995). *Rev. Sci. Instrum.* **66**, 1624–1626.

[bb10] Arnoldbik, W. M., Zeijlmans van Emmichoven, P. A. & Habraken, F. H. P. M. (2005). *Phys. Rev. Lett.* **94**, 245504.

[bb11] Arstila, K., Julin, J., Laitinen, M. I., Aalto, J., Konu, T., Kärkkäinen, S., Rahkonen, S., Raunio, M., Itkonen, J., Santanen, J.-P., Tuovinen, T. & Sajavaara, T. (2014). *Nucl. Instrum. Methods Phys. Res. Sect. B*, **331**, 34–41.

[bb12] Aumayr, F., Facsko, S., El-Said, A. S., Trautmann, C. & Schleberger, M. (2011). *J. Phys. Condens. Matter*, **23**, 393001. 10.1088/0953-8984/23/39/393001 21900733

[bb13] Awazu, K., Fujimaki, M., Ohki, Y. & Komatsubara, T. (2005). *Radiat. Meas.* **40**, 722–729.

[bb14] Awazu, K., Wang, X., Fujimaki, M., Komatsubara, T., Ikeda, T. & Ohki, Y. (2006). *J. Appl. Phys.* **100**, 044308.

[bb15] Bierschenk, T., Giulian, R., Afra, B., Rodriguez, M. D., Schauries, D., Mudie, S., Pakarinen, O. H., Djurabekova, F., Nordlund, K., Osmani, O., Medvedev, N., Rethfeld, B., Ridgway, M. C. & Kluth, P. (2013). *Phys. Rev. B*, **88**, 174111. 10.1103/PhysRevLett.110.245502 25165936

[bb16] Bogdanović-Radović, I., Buljan, M., Karlušić, M., Skukan, N., Božičević, I., Jakšić, M., Radić, N., Dražić, G. & Bernstorff, S. (2012). *Phys. Rev. B*, **86**, 165316.

[bb17] Bringa, E. M. & Johnson, R. E. (2002). *Phys. Rev. Lett.* **88**, 165501. 10.1103/PhysRevLett.88.165501 11955237

[bb18] Buljan, M., Bogdanović-Radović, I., Karlušić, M., Desnica, U. V., Dražić, G., Radić, N., Dubček, P., Salamon, K., Bernstorff, S. & Holý, V. (2009). *Appl. Phys. Lett.* **95**, 063104.

[bb19] Buljan, M., Bogdanović-Radović, I., Karlušić, M., Desnica, U. V., Radić, N., Jakšić, M., Salamon, K., Dražić, G., Bernstorff, S. & Holý, V. (2011). *Phys. Rev. B*, **84**, 155312.

[bb20] Buljan, M., Bogdanović-Radović, I., Karlušić, M., Desnica, U. V., Radić, N., Skukan, N., Dražić, G., Ivanda, M., Gamulin, O., Matej, Z., Valeš, V., Grenzer, J., Cornelius, T. W., Metzger, H. T. & Holý, V. (2010). *Phys. Rev. B*, **81**, 085321.

[bb21] Buljan, M., Karlušić, M., Bogdanović-Radović, I., Jakšić, M., Salamon, K., Bernstorff, S. & Radić, N. (2012). *Appl. Phys. Lett.* **101**, 103112.

[bb22] Buljan, M., Karlušić, M., Nekić, N., Jerčinović, M., Bogdanović-Radović, I., Bernsorff, S., Radić, N. & Mekterović, I. (2016). *Comput. Phys. Commun.* In the press.

[bb23] Buljan, M., Radić, N., Bernstorff, S., Dražić, G., Bogdanović-Radović, I. & Holý, V. (2012). *Acta Cryst.* A**68**, 124–138. 10.1107/S0108767311040104 PMC3243409 22186289

[bb24] Buljan, M. *et al.* (2015). *Nanotechnology*, **26**, 065602. 10.1088/0957-4484/26/6/065602 25605224

[bb25] Canut, B., Thevenard, P. & Jardin, C. (2004). *Nucl. Instrum. Methods Phys. Res. Sect. B*, **218**, 487–491.

[bb26] Cornelius, T. W., Schiedt, B., Severin, D., Pépy, G., Toulemonde, M., Apel, P. Y., Boesecke, P. & Trautmann, C. (2010). *Nanotechnology*, **21**, 155702. 10.1088/0957-4484/21/15/155702 20332555

[bb27] Eyal, Y. & Abu Saleh, S. (2007). *J. Appl. Cryst.* **40**, 71–76.

[bb28] Horcas, I., Fernández, R., Gómez-Rodríguez, J. M., Colchero, J., Gómez-Herrero, J. & Baro, A. M. (2007). *Rev. Sci. Instrum.* **78**, 013705. 10.1063/1.2432410 17503926

[bb29] Ishikawa, N., Okubo, N. & Taguchi, T. (2015). *Nanotechnology*, **26**, 355701. 10.1088/0957-4484/26/35/355701 26245538

[bb30] Itoh, N., Duffy, D. M., Khakshouri, S. & Stoneham, A. M. (2009). *J. Phys. Condens. Matter*, **21**, 474205. 10.1088/0953-8984/21/47/474205 21832484

[bb31] Jensen, J., Martin, D., Surpi, A. & Kubart, T. (2010). *Nucl. Instrum. Methods Phys. Res. Sect. B*, **268**, 1893–1898.

[bb32] Jensen, J., Skupiński, M., Hjort, K. & Sanz, R. (2008). *Nucl. Instrum. Methods Phys. Res. Sect. B*, **266**, 3113–3119.

[bb33] Karlušić, M., Akcöltekin, S., Osmani, O., Monnet, I., Lebius, H., Jakšić, M. & Schleberger, M. (2010). *New J. Phys.* **12**, 043009.

[bb34] Karlušić, M. & Jakšić, M. (2012). *Nucl. Instrum. Methods Phys. Res. Sect. B*, **280**, 103–110.

[bb35] Karlušić, M., Kozubek, R., Lebius, H., Ban-d’Etat, B., Wilhelm, R. A., Buljan, M., Siketić, Z., Scholz, F., Meisch, T., Jakšić, M., Bernstorff, S., Schleberger, M. & Šantić, B. (2015). *J. Phys. D Appl. Phys.* **48**, 325304.

[bb36] Kluth, P., Schnohr, C. S., Pakarinen, O. H., Djurabekova, F., Sprouster, D. J., Giulian, R., Ridgway, M. C., Byrne, A. P., Trautmann, C., Cookson, D. J., Nordlund, K. & Toulemonde, M. (2008). *Phys. Rev. Lett.* **101**, 175503. 10.1103/PhysRevLett.101.175503 18999762

[bb37] Kuttich, B., Engel, M., Trautmann, C. & Stühn, B. (2014). *Appl. Phys. A*, **114**, 387–392.

[bb38] Mayer, M. (1997). *SIMNRA User’s Guide*. Report IPP 9/113, Max-Planck-Institut für Plasmaphysik, Garching, Germany.

[bb39] Nomura, K., Nakanishi, T., Nagasawa, Y., Ohki, Y., Awazu, K., Fujimaki, M., Kobayashi, N., Ishii, S. & Shima, K. (2003). *Phys. Rev. B*, **68**, 064106.

[bb40] Ochedowski, O., Kleine Bussmann, B., Ban-d’Etat, B., Lebius, H. & Schleberger, M. (2013). *Appl. Phys. Lett.* **102**, 153103.

[bb41] Ochedowski, O., Lehtinen, O., Kaiser, U., Turchanin, A., Ban-d’Etat, B., Lebius, H., Karlušić, M., Jakšić, M. & Schleberger, M. (2015). *Nanotechnology*, **26**, 465302. 10.1088/0957-4484/26/46/465302 26510213

[bb42] Ochedowski, O., Osmani, O., Schade, M., Bussmann, B. K., Ban-d’Etat, B., Lebius, H. & Schleberger, M. (2014). *Nat. Commun.* **5**, 3913. 10.1038/ncomms4913 24905053

[bb43] Papaléo, R. M., Silva, M. R., Leal, R., Grande, P. L., Roth, M., Schattat, B. & Schiwietz, G. (2008). *Phys. Rev. Lett.* **101**, 167601. 10.1103/PhysRevLett.101.167601 18999714

[bb44] Papaléo, R. M., Thomaz, R., Gutierres, L. I., de Menezes, V. M., Severin, D., Trautmann, C., Tramontina, D., Bringa, E. M. & Grande, P. L. (2015). *Phys. Rev. Lett.* **114**, 118302. 10.1103/PhysRevLett.114.118302 25839315

[bb45] Pépy, G., Boesecke, P., Kuklin, A., Manceau, E., Schiedt, B., Siwy, Z., Toulemonde, M. & Trautmann, C. (2007). *J. Appl. Cryst.* **40**, s388–s392.

[bb46] Popok, V. N., Jensen, J., Vučković, S., Mackova, A. & Trautmann, C. (2009). *J. Phys. D Appl. Phys.* **42**, 205303.

[bb47] Ridgway, M. C. *et al.* (2013). *Phys. Rev. Lett.* **110**, 245502.

[bb48] Rivera, A., Crespillo, M. L., Olivares, J., Sanz, R., Jensen, J. & Agulló-López, F. (2010). *Nucl. Instrum. Methods Phys. Res. Sect. B*, **268**, 3122–3126.

[bb49] Rodríguez, M. D., Afra, B., Trautmann, C., Toulemonde, M., Bierschenk, T., Leslie, J., Giulian, R., Kirby, N. & Kluth, P. (2012). *J. Non-Cryst. Solids*, **358**, 571–576.

[bb50] Roll, T., Meier, M., Akcöltekin, S., Klusmann, M., Lebius, H. & Schleberger, M. (2008). *Phys. Status Solidi (RRL)*, **2**, 209–211.

[bb54] Sanz, R., Jaafar, M., Hernández-Vélez, M., Asenjo, A., Vázquez, M. & Jensen, J. (2010). *Nanotechnology*, **21**, 235301. 10.1088/0957-4484/21/23/235301 20463385

[bb55] Sanz, R., Jensen, J., Johansson, A., Skupinski, M., Possnert, G., Boman, M., Hernandez-Vélez, M., Vázquez, M. & Hjort, K. (2007). *Nanotechnology*, **18**, 305303.

[bb56] Sanz, R., Johansson, A., Skupinski, M., Jensen, J., Possnert, G., Boman, M., Vázquez, M. & Hjort, K. (2006). *Nano Lett.* **6**, 1065–1068.

[bb57] Schattat, B., Bolse, W., Klaumünzer, S., Zizak, I. & Scholz, R. (2005). *Appl. Phys. Lett.* **87**, 173110.

[bb58] Schauries, D., Afra, B., Rodriguez, M. D., Trautmann, C., Hawley, A. & Kluth, P. (2016). *Nucl. Instrum. Methods Phys. Res. Sect. B*, **365A**, 380–383.

[bb59] Schauries, D., Lang, M., Pakarinen, O. H., Botis, S., Afra, B., Rodriguez, M. D., Djurabekova, F., Nordlund, K., Severin, D., Bender, M., Li, W. X., Trautmann, C., Ewing, R. C., Kirby, N. & Kluth, P. (2013). *J. Appl. Cryst.* **46**, 1558–1563.

[bb60] Schwartz, K., Trautmann, C., Steckenreiter, T., Geiß, O. & Krämer, M. (1998). *Phys. Rev. B*, **58**, 11232–11240.

[bb61] Semenyuk, A. V., Svergun, D. I., Mogilevsky, L. Yu., Berezkin, V. V., Mchedlishvili, B. V. & Vasilev, A. B. (1991). *J. Appl. Cryst.* **24**, 809–810.

[bb62] Siketić, Z., Bogdanović Radović, I. B. & Jakšić, M. (2008). *Nucl. Instrum. Methods Phys. Res. Sect. B*, **266**, 1328–1332.

[bb63] Siketić, Z., Bogdanović Radović, I. B. & Jakšić, M. (2010). *Thin Solid Films*, **518**, 2617–2622.

[bb64] Siketić, Z., Bogdanović Radović, I. B., Jakšić, M. & Skukan, N. (2010). *Rev. Sci. Instrum.* **81**, 033305. 10.1063/1.3356976 20370168

[bb65] Siketić, Z., Skukan, N. & Bogdanović Radović, I. (2015). *Rev. Sci. Instrum.* **86**, 083301. 10.1063/1.4927605 26329175

[bb66] Thevenard, P. A., Dupin, J.-P., Vu Thien, B., Purcell, S. T. & Semet, V. (2000). *Surf. Coat. Technol.* **128–129**, 59–65.

[bb67] Toulemonde, M., Assmann, W., Dufour, C., Meftah, A. & Trautmann, C. (2012). *Nucl. Instrum. Methods Phys. Res. Sect. B*, **277**, 28–39.

[bb68] Toulemonde, M., Assmann, W., Trautmann, C. & Grüner, F. (2002). *Phys. Rev. Lett.* **88**, 057602. 10.1103/PhysRevLett.88.057602 11863780

[bb69] Toulemonde, M., Trautmann, C., Balanzat, E., Hjort, K. & Weidinger, A. (2004). *Nucl. Instrum. Methods Phys. Res. Sect. B*, **216**, 1–8.

[bb70] Trautmann, C., Toulemonde, M., Schwartz, K., Costantini, J. M. & Müller, A. (2000). *Nucl. Instrum. Methods Phys. Res. Sect. B*, **164–165**, 365–376.

[bb71] Zhang, Y., Debelle, A., Boulle, A., Kluth, P. & Tuomisto, F. (2015). *Curr. Opin. Solid State Mater. Sci.* **19**, 19–28.

[bb72] Ziegler, J. F., Ziegler, M. D. & Biersack, J. P. (2010). *Nucl. Instrum. Methods Phys. Res. Sect. B*, **268**, 1818–1823.

